# Correction: Novel insights into the morphology of *Plesiochelys bigleri* from the early Kimmeridgian of Northwestern Switzerland

**DOI:** 10.1371/journal.pone.0227509

**Published:** 2019-12-30

**Authors:** Irena Raselli, Jérémy Anquetin

The Funding statement is incorrect. The correct funding statement is as follows: This work was supported by the Swiss National Science Foundation (SNF 205321_175978).

The captions for Fig 1 and Fig 2 are switched. Please see the correct captions here.

**Fig 1:**
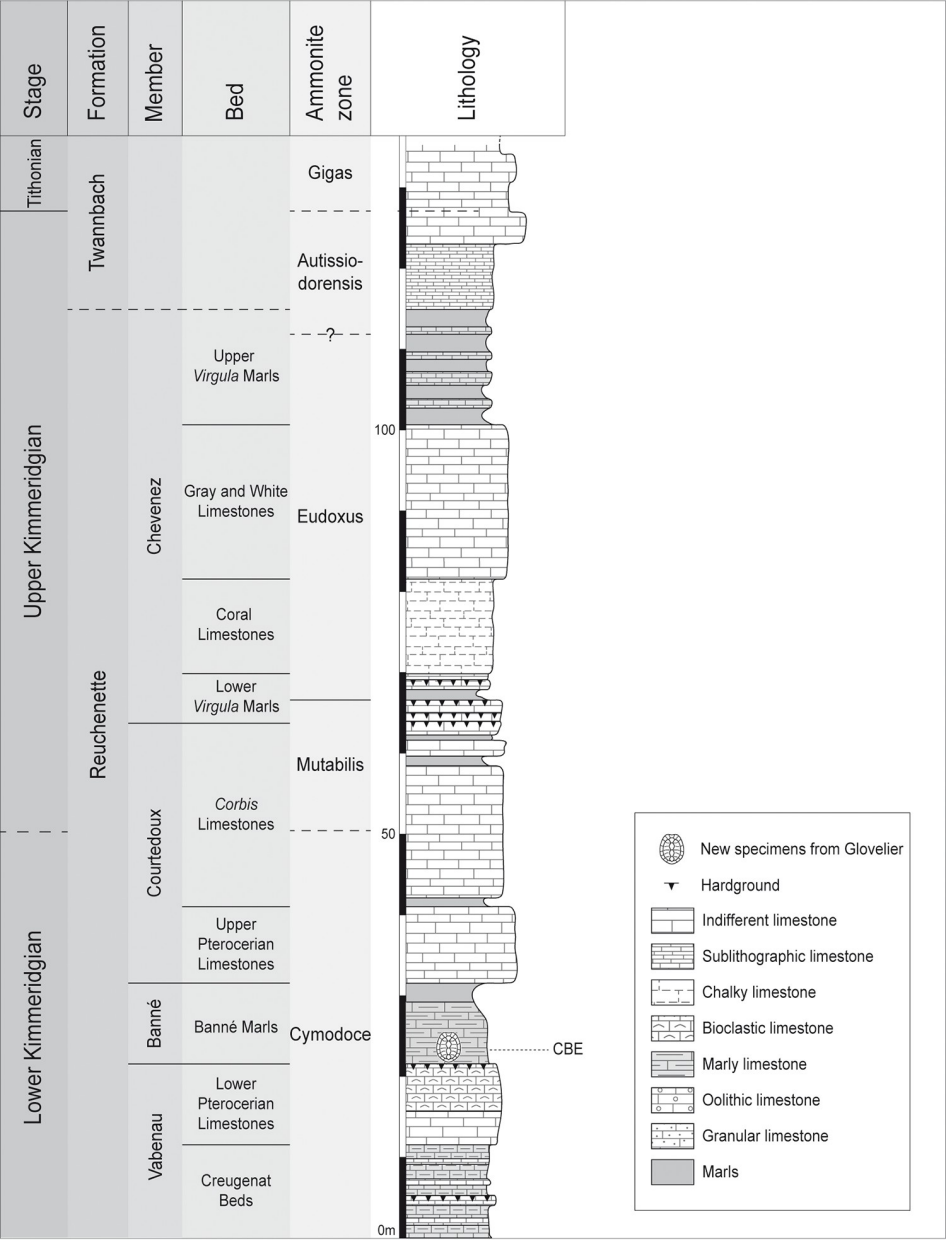
Stratigraphical column of the Reuchenette Formation in northwestern Switzerland. The shell marks the position of the new specimens in the Banné Marls (CBE: Combe du Bé, name of the locality). Modified from Püntener et al. [11].

**Fig 2:**
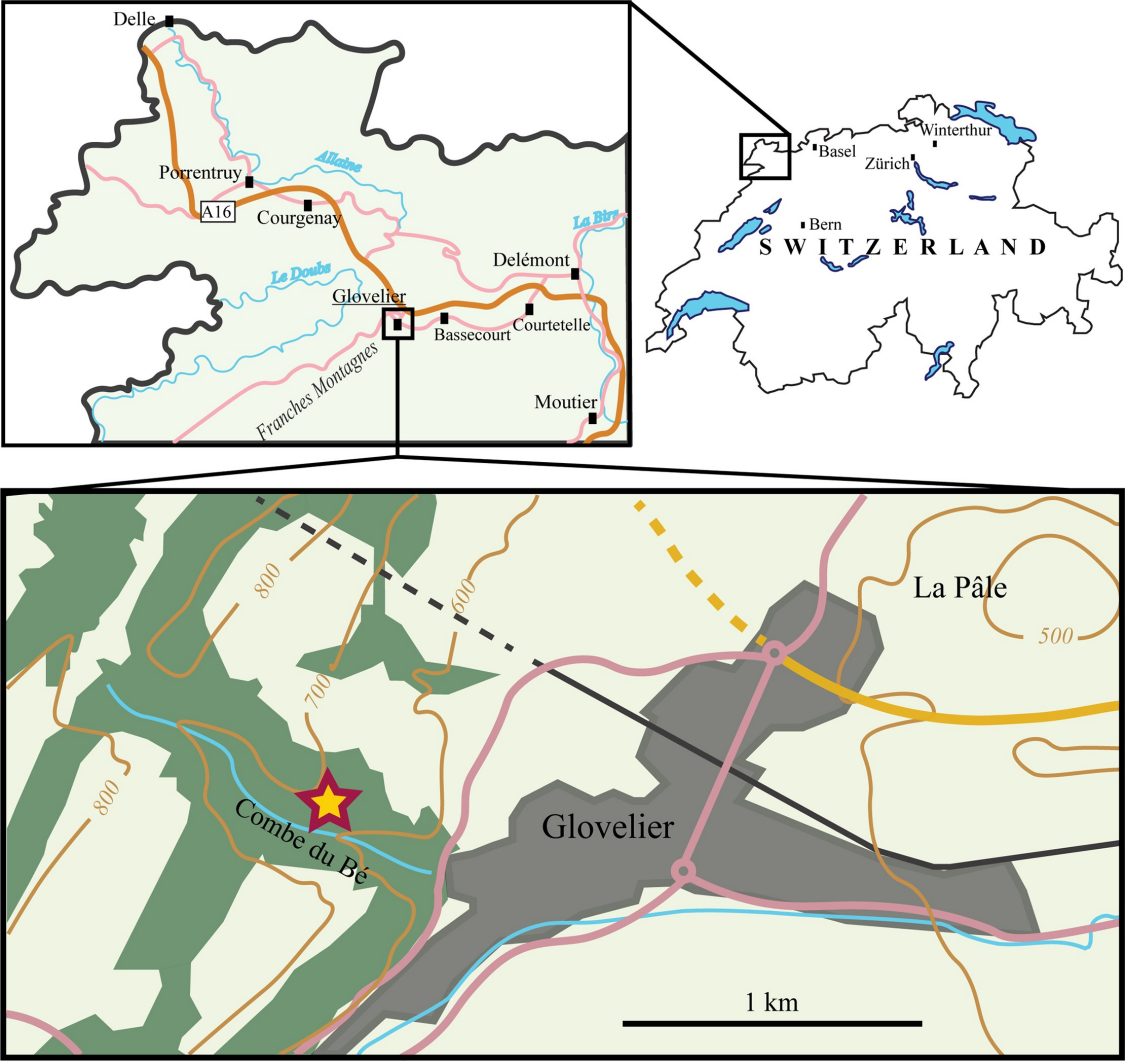
Map of the locality. The star marks the location of the outcrop in the small valley Combe du Bé near Glovelier, Canton of Jura, northwestern Switzerland.
