# Supplementary figures and images for: Novel insights into the morphology of Plesiochelys bigleri from the early Kimmeridgian of Northwestern Switzerland
Source: PLoS One. 2019 May 15;14(5):e0214629. doi: 10.1371/journal.pone.0214629 (PMC6519798; doi:10.1371/journal.pone.0214629)

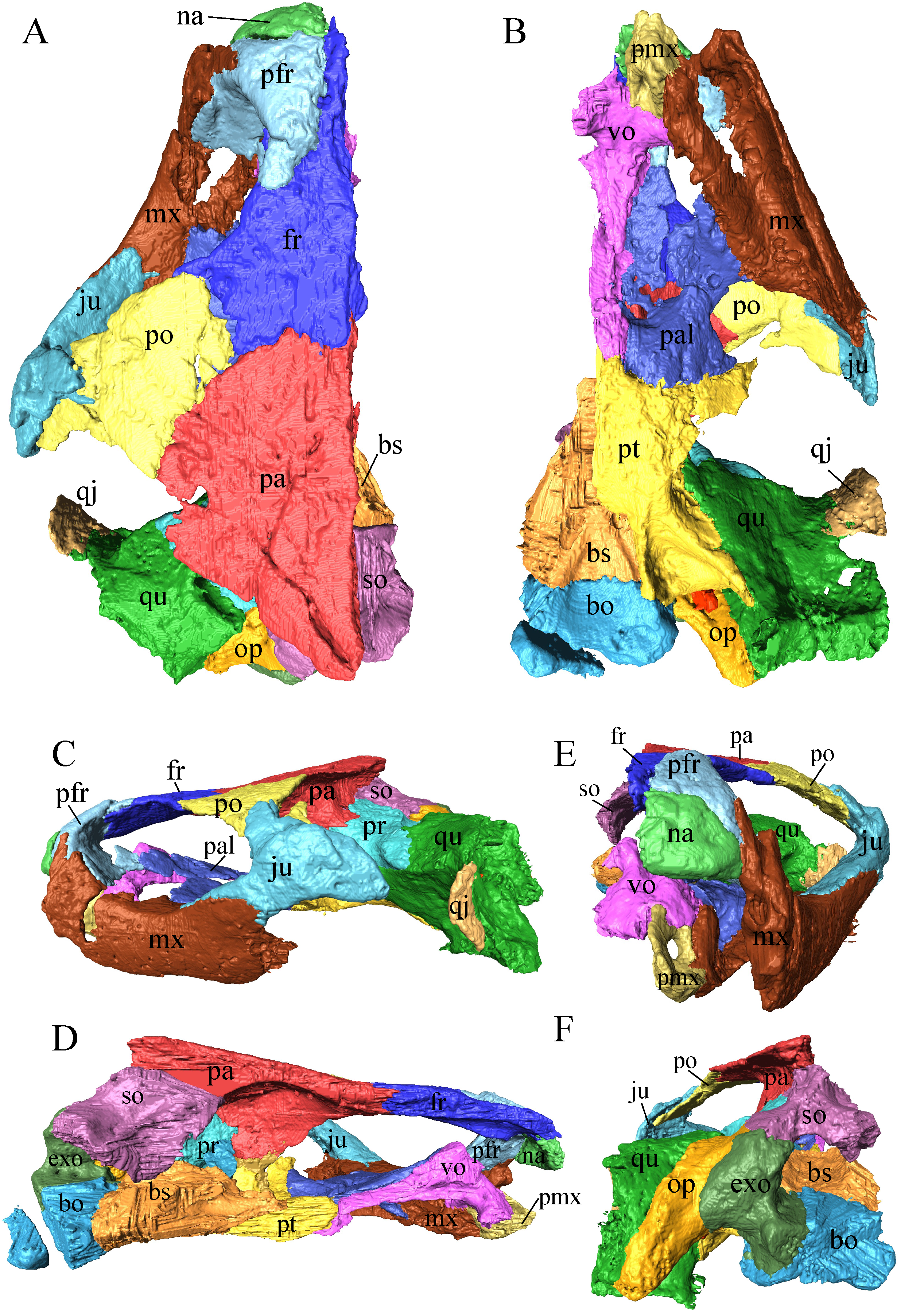

Supplement: S1 Fig — Views: dorsal (A), ventral (B), anterior (C), posterior (D), medial (E), lateral (F). (TIF) [file pone.0214629.s001.tif]

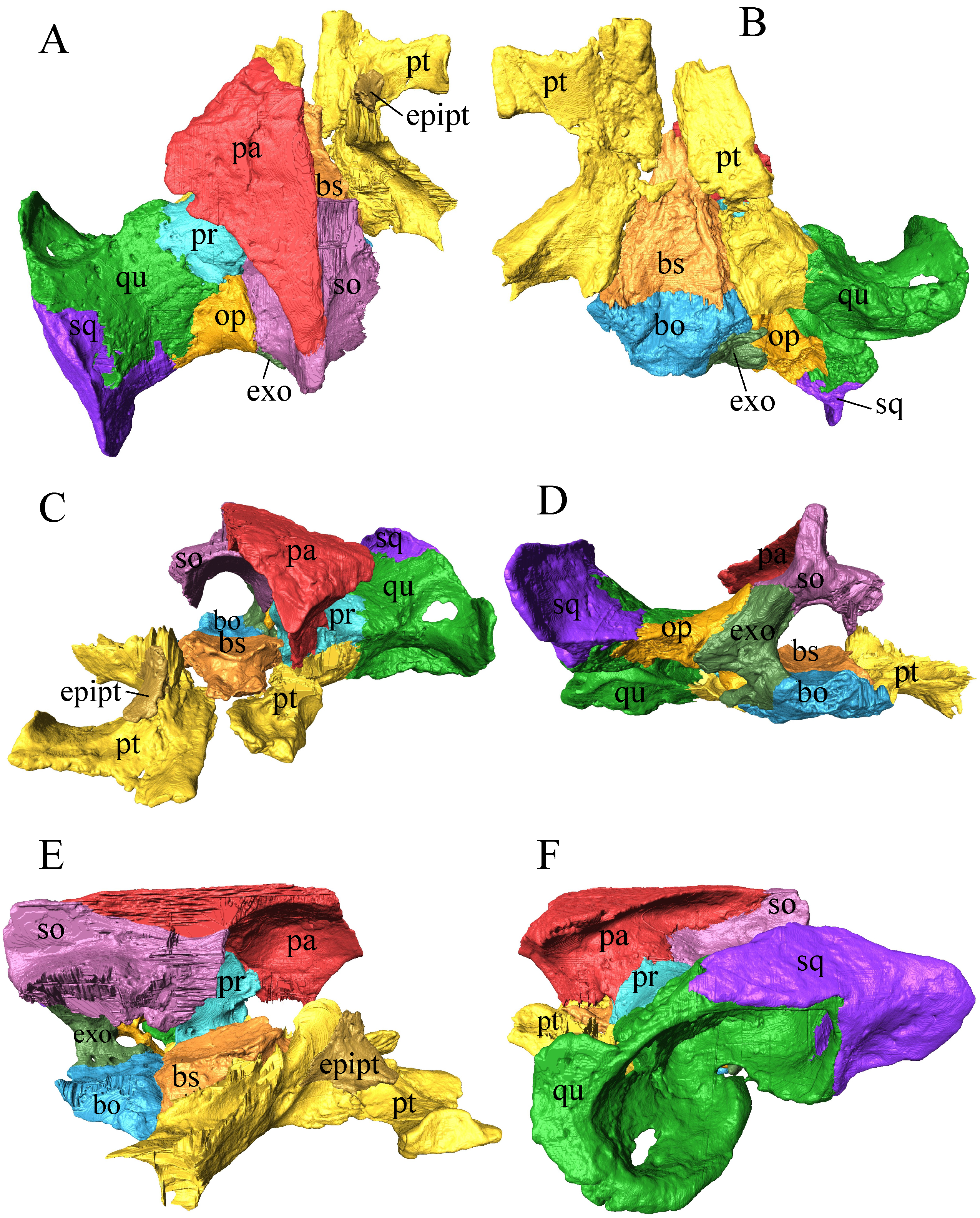

Supplement: S2 Fig — Views: A: dorsal, B: ventral, C: anterior, D: posterior, E: medial, F: lateral. (TIF) [file pone.0214629.s002.tif]
